# Recurrent Infective Endocarditis With Recurrent Intravenous Drug Use: Illustrating the High Complexity of Surgical Decision-Making

**DOI:** 10.1016/j.atssr.2025.11.032

**Published:** 2026-01-22

**Authors:** Haris Patail, Yasser Radwan, Hasan Ahmad, Junichi Shimamura, Suguru Ohira, Syed Abbas Haidry, Joshua Goldberg, Daniel M. Spevack

**Affiliations:** 1 Department of Cardiology, Westchester Medical Center, Valhalla, New York; 2 Department of Cardiac Surgery, Westchester Medical Center, Valhalla, New York; 3 Department of Cardiac Surgery, Hartford Hospital, Hartford, Connecticut; 4 Department of Cardiothoracic Surgery, Weill Cornell Medical College, New York, New York

## Abstract

Management of infective endocarditis in patients who inject drugs is often a significant challenge. The decision to proceed with surgical management frequently occurs in patients experiencing a complex web of psychosocial, socioeconomic, medical, and legal issues that can be difficult for clinicians to address. The recent addition of percutaneous mechanical aspiration as a potential adjunctive tool adds further still to the complexity of picking a rational therapeutic strategy. We report a case of recurrent infective endocarditis in a patient who injects drugs that illustrates the many difficult dilemmas commonly presented in this population.

The United States is currently in a fourth wave of an opioid epidemic, and the annual incidence of infective endocarditis has paralleled the near-exponential rise in use of intravenous drugs.^1^ Current guidelines advocate for surgery when there is valve dysfunction resulting in clinical heart failure, persistent bacteremia with resistant organisms despite appropriate antibiotic therapy, or evidence of an embolic phenomenon.^2^ Because most infective endocarditis cases related to patients who inject drugs (PWID) involve right-sided heart valves with high recurrence rates, guidelines recommend more conservative, nonsurgical strategies to prove effective before resorting to surgery.^2^ Although essential, prolonged intravenous antibiotic therapy can also present significant challenges in this population. Accordingly, percutaneous mechanical aspiration (PMA) has emerged as a potential option to help achieve source control by debulking vegetations.^3^ Several studies have demonstrated that this strategy is safe with good results; however, there is a lack of randomized controlled trials comparing its effectiveness to traditional practice standards.^3^

A 28-year-old man with a history of intravenous drug use (IVDU) underwent 5 tricuspid valve (TV) replacements and 3 PMA procedures for TV endocarditis during a 7-year period between 2016 and 2023 (Figure 1). His opioid use began with physician-prescribed medications progressing to IVDU. During each admission with TV endocarditis, multidisciplinary meetings were held to discuss management strategies, which included at least 1 cardiac surgeon, an interventional cardiologist, and a noninvasive cardiologist. As depicted in Figure 1, the decision to use PMA initially in April 2022 was made as an attempt to avoid recurrent TV surgery in a patient who continued to use drugs with a prior history of multiple surgeries. When he had recurrence 4 months later with the same organism, more definitive surgical management was thought to be necessary. In April 2023, the patient presented with extremely large vegetations and severe valve regurgitation. PMA was employed as a debulking procedure before planned valve replacement 1 week later. In September 2023, the patient presented with a *Candida* infection several months after he had ceased IVDU. He had lost weight, was frail appearing, and was believed to be too high risk for a surgical approach. PMA was again employed successfully with cure. When he had his final recurrence in November 2023, it was with yet another organism. He had regained weight and underwent successful curative valve replacement. Figure 2 shows transesophageal and fluoroscopy views of intraprocedural PMA for TV infective endocarditis from his first admission.

**Figure 1:**
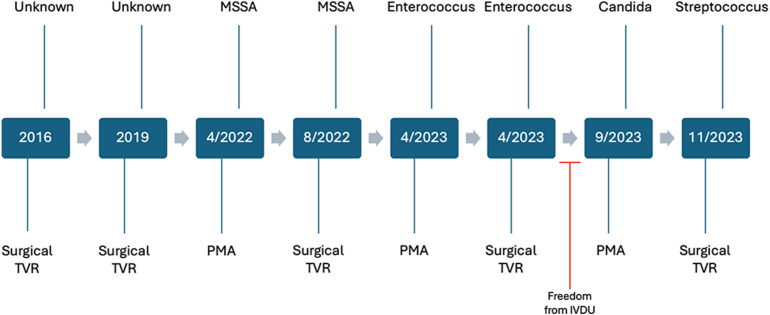
Diagram illustration of timeline between percutaneous mechanical aspiration (PMA) and tricuspid valve replacement (TVR). (IVDU, intravenous drug use; MSSA, methicillin-sensitive *Staphylococcus aureus*.)

**Figure 2:**
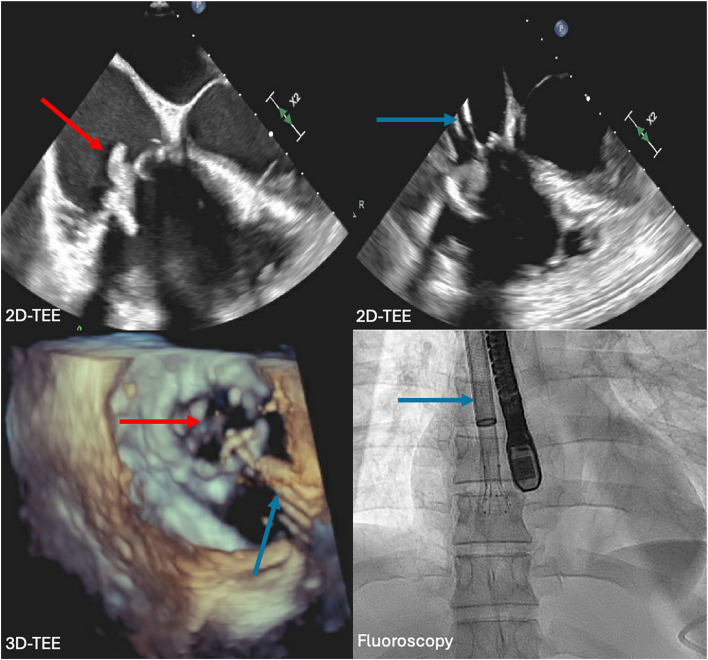
Transesophageal echocardiography (TEE), 2-dimensional (2D) and 3-dimensional (3D), and fluoroscopy images during percutaneous mechanical aspiration for tricuspid valve intervention. The red arrows indicate vegetation and the blue arrows indicate percutaneous suction cannula.

His admissions included prolonged hospital stays for >6 weeks, allowing successful completion of intravenous antibiotics. He reported a prior experience of home intravenous antibiotics through a central catheter after his initial valve surgery, which had been performed at a different institution. During admissions, he also received treatment of opioid addiction in both the inpatient and outpatient setting as part of a comprehensive program. Since the spring of 2023, the patient reports that he has not used intravenous drugs, supported by urine toxicology. The patient has continued to be followed up as an outpatient and has been free of IVDU and recurrent infection.

## Comment

In treating endocarditis in PWID, the decision to pursue surgical management is often complicated by concerns of potential postoperative reinfection and the ability of the patient to comply with prolonged intravenous antibiotic courses.^4^ These concerns must be weighed against the possibility of a surgical cure, which has the potential to be lifesaving. Prior data have shown that reinfection rates are much higher in this population, including reoperation rates that approached nearly 10 times the rates seen in non-PWID individuals.^4^^,^^5^

**Figure 3:**
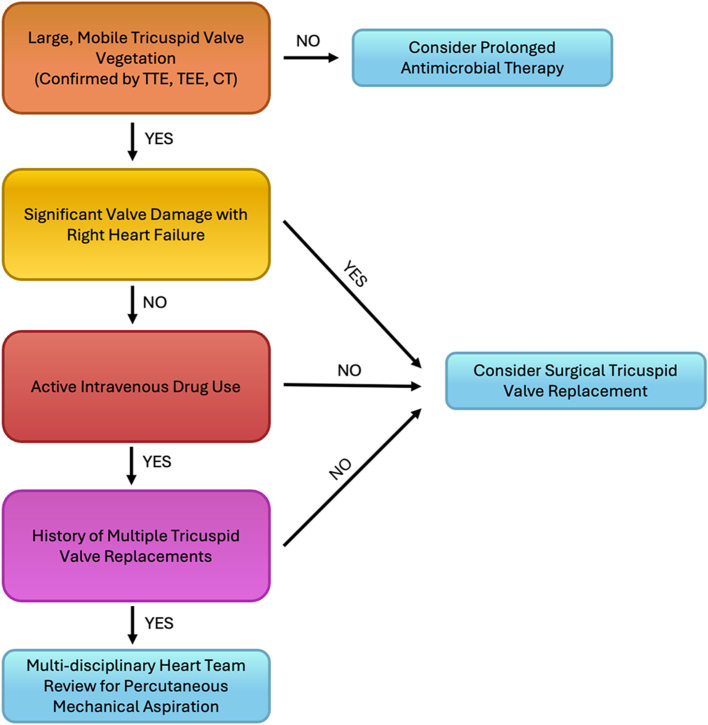
Treatment strategy guideline for opting for surgical tricuspid valve intervention, percutaneous mechanical aspiration, or medical therapy alone. (CT, computed tomography; TEE, transesophageal echocardiography; TTE, transthoracic echocardiography.)

PMA has recently been introduced as a potential therapeutic approach for endocarditis in PWID.^3^ A percutaneous approach can offer improved source control over medical management with less morbidity and cost compared with surgery.^3^ Retrospective studies have shown that PMA can be performed safely and with good outcomes in selected patients.^3^ Although prone to selection bias, observational studies have also compared outcomes in patients chosen for surgical strategies with those in patients chosen for PMA or medical therapy alone.^6^ Figure 3 depicts a treatment strategy guideline for opting for surgical TV intervention, PMA, or medical therapy alone.

Regardless of the treatment strategy chosen to achieve source control, all patients with endocarditis are recommended to receive prolonged courses of antibiotics. Many practical concerns make this crucial aspect of endocarditis treatment particularly challenging in the population of PWID. The American Heart Association guideline statement from 2022 offers an algorithm to help select which patients would be most likely to safely receive home intravenous antibiotics.^7^ Oral antibiotic regimens can also be initiated in select patients.^7^

As mentioned in our case summary, concerns about the ethics of offering repeated surgical or PMA interventions in PWID are likely to arise. Arguments regarding treatment futility and resource allocation are frequently cited as rational to avoid repeated interventions. Ethicist Daniel Daly argues against using such reasoning, invoking a principlist approach.^8^ Principlism is an ethical framework that invokes 4 core principles to guide moral decision-making.^8^ The principles of autonomy, beneficence, non-maleficence, and justice are cited as long agreed on as central to medical ethics.^8^ When he applies these principles to an individual who injects drugs, he argues that if the considered intervention is more likely than not to achieve its short-term objective to cure the infection, it should not be denied to an individual who desires to undergo the therapy.^8^ He states, “Medical professionals can and should distribute medical treatments based on the probability that the treatment will be medically effective. Statistics regarding recidivism post valve replacement provide insight regarding past patients’ behaviors, but such data are not predictive of whether this patient will return. Medical providers who attempt to determine psychosocial and qualitative futility commit the error of generalizing their expertise.”^8^

Our case highlights a challenging scenario of a young patient with recurrent bioprosthetic TV endocarditis in the setting of ongoing IVDU. In this case, the use of repeated surgery and PMA along with prolonged antibiotic therapy led to his reaching a period of stability without IVDU. Whereas an individual case with a positive outcome does not necessarily justify proceeding with similar management, it does help reinforce the notion that with support, some patients are able to quit opioid use and to break the cycle of recurrent infections.
